# Increasing incidence of invasive and in situ cervical adenocarcinoma in the Netherlands during 2004–2013

**DOI:** 10.1002/cam4.971

**Published:** 2017-01-19

**Authors:** Judith van der Horst, Albert G. Siebers, Johan Bulten, Leon F. Massuger, Inge MCM de Kok

**Affiliations:** ^1^ Departments of PathologyRadboud University Medical CenterNijmegenThe Netherlands; ^2^ Department of Obstetrics and GynaecologyRadboud University Medical CenterNijmegenThe Netherlands; ^3^ Department of Public HealthErasmus MCUniversity Medical CenterRotterdamThe Netherlands

**Keywords:** Adenocarcinoma in situ, carcinoma, cervix, HPV, incidence

## Abstract

In the developed world, the incidence of cervical squamous cell carcinoma has decreased, however, the incidence of adenocarcinoma in situ (AIS) and invasive adenocarcinoma increased, predominantly in young females. The goal of this study was to evaluate the most recent incidence rates of AIS, adenocarcinoma, and squamous cell carcinoma of the uterine cervix in the Netherlands in 2004–2013. By using Dutch national pathology and cancer registries, we calculated European standardized incidence rates (ESR) and estimated annual percentage changes (EAPC) for AIS during 2004–2013 and for invasive cervical carcinomas during 1989–2013. For AIS, presence or absence of concomitant cervical intraepithelial neoplasia (CIN) was explored. The estimated annual percentage change (EAPC) of squamous cell carcinoma decreased significantly in 1989–2013, predominantly in 1989–2003. The EAPC of invasive adenocarcinoma decreased in 1989–2003, but remained stable in 2004–2013. The EAPC of AIS increased significantly, predominantly in women of 25–39 years old. Of these AIS cases, 58.9% had concomitant CIN and AIS with concomitant CIN showed a significantly higher EAPC compared to AIS without CIN. Our conclusion is that despite a nationwide screening program for cancer of the uterine cervix, the incidence of adenocarcinoma in the Netherlands remained stable during 2004–2013 and the incidence of adenocarcinoma in situ increased. This was most predominant in cases with concomitant CIN and in younger females. The incidence of squamous cell carcinoma decreased in the same timeframe.

## Introduction

Cervical cancer is the second most common cancer and the third most common cause of cancer‐related death in females worldwide. Most cervical cancers are squamous cell carcinomas. Adenocarcinoma of the uterine cervix accounts worldwide for 10–20% of all cervical cancers 1. Adenocarcinoma in situ (AIS) of the uterine cervix is considered as a precancerous lesion of invasive cervical adenocarcinoma 2. It has been shown that adenocarcinoma, compared to squamous cell carcinoma with the same stage and tumor size, has a poorer prognosis due to a higher rate of metastases 3.

In high‐income countries such as the USA and European countries, there is a trend of decreasing incidence of squamous cell carcinoma, but a relative and absolute increase in the incidence of adenocarcinoma of the uterine cervix 1, 4, 5, 6, 7. Most studies found that this increase was caused by an increasing incidence in younger women (under 55 years). In the Netherlands, similar trends in incidence of invasive cervical carcinomas were found. The incidence and mortality rates of squamous cell carcinoma have decreased in past decades 8 only to increase in the last decade 9. The incidence of cervical adenocarcinoma remained relatively stable or increased, predominantly caused by an increasing incidence in younger women 8. This differed for the precursor lesions as in 1987–2003 a decreasing incidence of adenocarcinoma in situ (AIS) was found 10.

Because there are no recent rates for the Netherlands, the aim of this study was to explore the most recent incidence rates of AIS, adenocarcinoma, and squamous cell carcinoma of the uterine cervix in the Netherlands. Changes in these rates can be an indication of alterations in underlying risk factors and/ or screening procedures. The correlation between AIS and the presence or absence of concomitant cervical intraepithelial neoplasia (CIN) was also investigated.

## Methods

### Data collection

Data concerning the incidence of adenocarcinoma, squamous cell carcinoma, and other malignant cancers of the uterine cervix were obtained from the Netherlands national cancer registry (IKNL, www.cijfersoverkanker.nl). Cancers such as adenosquamous carcinoma, adenoid cystic carcinoma, and small‐cell carcinoma were grouped together and classified as “other” malignant cancers. Data covering the years 1989–2013 were collected. Data concerning the population size were retrieved from the website of Statistics Netherlands (statline.cbs.nl).

Since the national cancer registry only registers the invasive carcinomas, data concerning AIS had to be retrieved from “the nationwide network and registry of histo‐ and cytopathology in the Netherlands” (PALGA). In this nationwide network, excerpts of all histopathology and cytopathology reports are generated automatically at the pathology laboratories and transferred to a central database, with nationwide coverage from 1991 onwards 11. Patients can object to the uptake of their information in this database. The excerpts from the database comprise a free text field for the full conclusion and a SNOMED‐like coded diagnosis line, describing the type of material and the histological diagnosis. Patients are identified through their birth date and the first eight letters of their (maiden) family name. This identification code enables linkage of the patient's tests, preventing double inclusion. Any request for information for scientific research from this database has to be approved by the Pathologisch Anatomisch Landelijk Geautomatiseerd Archief (PALGA) committee and information is coded so it is not directly convertible to specific patients. For the period 2004–2013, all histological records with “adenocarcinoma”, “adenocarcinoma in situ”, and/or “atypia” mentioned in the conclusion or diagnosis‐line, combined with the term cervix, were selected. A total of 9900 records from 6138 patients were retrieved. These records were scrutinized for the actual presence of the diagnosis adenocarcinoma in situ, CGIN3 (cervical glandular intraepithelial neoplasia grade 3), and severe or high‐grade atypia of the endocervical cylindrical cells. Patients were excluded if there was only information available concerning cytology, when it was not clear if the lesion was glandular in origin, if there was only mild or moderate atypia of the endocervical cells, when the lesion was described as adenosquamous or when the lesion was not primarily cervical in origin, but for instance endometrial. Cases with (micro)invasive growth were also excluded. Age was defined as the age at primary diagnosis of AIS. Furthermore, the presence of concomitant CIN, ranging from CIN1 to invasive squamous carcinoma was assessed. Data concerning racial/ethnic groups are not available in the database, so we cannot comment on racial/ethnic differences.

### Statistical analysis

Crude rates were calculated per 10^5^ women years. For invasive carcinomas, absolute incidence rates from the national cancer registry were used. Age‐adjusted rates were calculated for the European standard population per 10^5^ women years resulting in European Standardized Rates (ESR). The ESR was used to calculate the Estimated Annual Percentage Change (EAPC) by transforming all ESR into a natural logarithm (ln) and using calendar year as a regression variable to fit a regression line to the natural logarithms of the ESR using calendar year as independent variable. The formula that was used was y = mx + b, where y = ln(ESR) and x = calendar year. Then, EAPC equals 100*(e^m^−1). The standard error is obtained from the fit of the regression line and used to calculate the 95% confidence interval, again using the above mentioned formula. The null‐hypothesis was that the slope of the curve is zero. The statistical difference of the incidence rates over time from the null‐hypothesis was calculated using Pearson's correlation coefficient with two‐sided significance. The differences in percentages and age for AIS with and without concomitant squamous lesions were calculated with *t*‐test. All analysis was performed using IBM SPSS statistical software (version 20.0.01).

## Results

**Figure 1:**
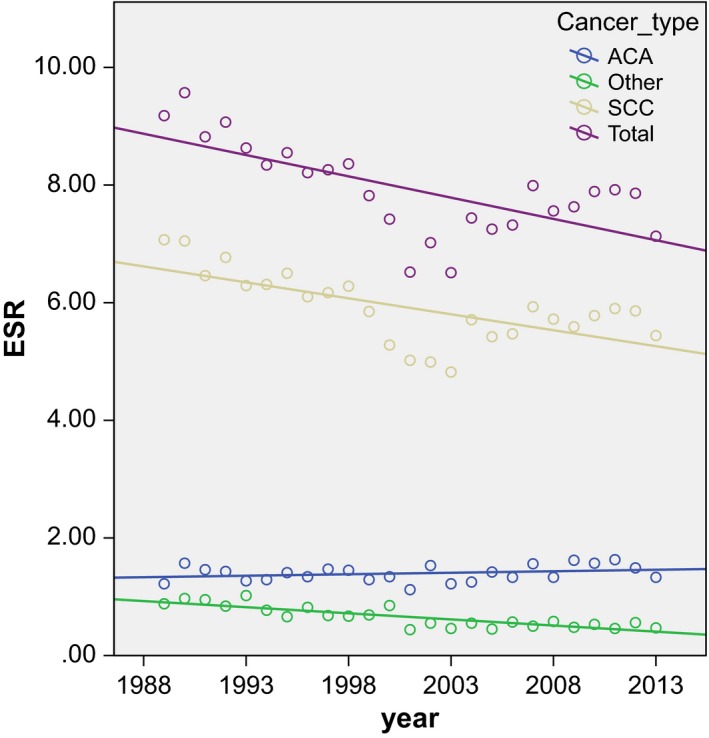
Scatterplot of the European standardized rates (ESR) of all cervical cancer and the different cancer types with correlation line in the period 1989–2013. (Data extracted from www.cijfersoverkanker.nl).

Figure 1 shows the European standardized rates of the three different cervical cancer types and of all cervical cancer in the period 1989–2013. In Table 1, the estimated annual percentage change of the three subgroups and of all cervical cancer in the periods 1989–2013, 1989–2003, and 2004–2013 is shown. There was a significant decrease in the incidence of squamous cell carcinoma of the uterine cervix (*r *= −0.67; *P* = 0.00) with an EAPC of −0.90% (95% CI:−1.28, −0.51; *P* = 0.00) and of other cervical carcinomas (including adenosquamous carcinoma) (*r* = −0.84; *P* = 0.00) with an EAPC of −3.05% (95%CI: −3.81, −2.29; *P* = 0.00) in the Netherlands in the period 1989–2013. This decrease in squamous cell carcinomas is predominantly caused by the age groups of 60+ years (data not shown). The incidence of adenocarcinoma of the uterine cervix remained relatively stable (*r* = 0.27; *P* = 0.19) with an EAPC of 0.40% (95% CI: −0.19, 0.99; *P* = 0.21) in this same period. Overall, the incidence of cervical cancer decreased in 1989 to 2013 (*r* = −0.68; *P* = 0.00) with an EAPC of −0.90% (95% CI: −1.28, −0.51; *P* = 0.00). However, when the rates are subdivided into two time periods, the EAPC of all cancer types, including adenocarcinoma, was negative in 1989–2003, although the change is nonsignificant for this last group. During 2004–2013, the EAPC for all cancer types remained stable. All three cancer types show two peaks in incidence: the first at 35–39 years and the second at 70–74 years age (data not shown in figures).

**Table 1:** EAPC, 95% confidence interval (95% CI) and ***P***‐value for SCC, ACA, other cervical carcinomas (other), and total cervical carcinomas (total) in the periods 1989–2013, 1989–2003, and 2004–2013. (Data extracted from www.cijfersoverkanker.nl)

	1989–2013			1989–2003			2004–2013		
	EAPC (%)	95% CI (%)	*P*‐value	EAPC (%)	95% CI (%)	*P*‐value	EAPC (%)	95% CI (%)	*P*‐value
SCC	−0.90	−1.28, −0.51	0.00	−2.57	−3.14, −1.99	0.00	0.20	−0.59, 0.99	0.56
ACA	0.40	−0.19, 0.99	0.21	−0.50	−1.66, 0.68	0.39	1.31	−0.66, 3.31	0.24
Other	−3.05	−3.81, −2.29	0.00	−4.59	−6.26, −2.89	0.00	−0.60	−2.72, 1.57	0.61
Total	−0.90	−1.28, −0.51	0.00	−2.47	−3.04, −1.89	0.00	0.30	−0.68, 1.29	0.49

EAPC, estimated annual percentage change; SCC, squamous cell carcinoma**;** ACA; adenocarcinoma.

**Figure 2:**
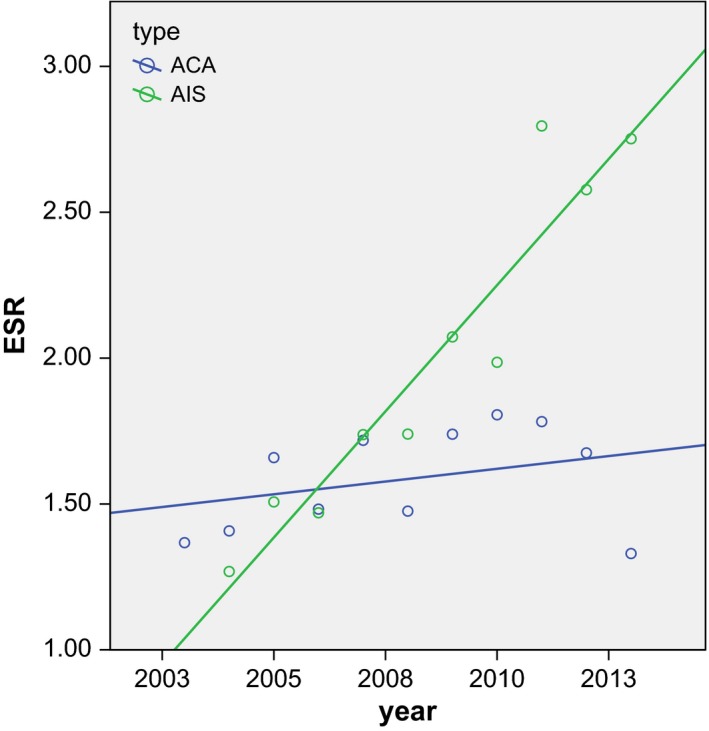
Scatterplot of the European Standardized Rates of AIS in the years 2004–2013 with correlation lines. ACA *r* = 0.330 with *P* = 0.322/AIS *r* = 0.951 with *P* = 0.000. ACA, adenocarcinoma; AIS, adenocarcinoma in situ.

**Figure 3:**
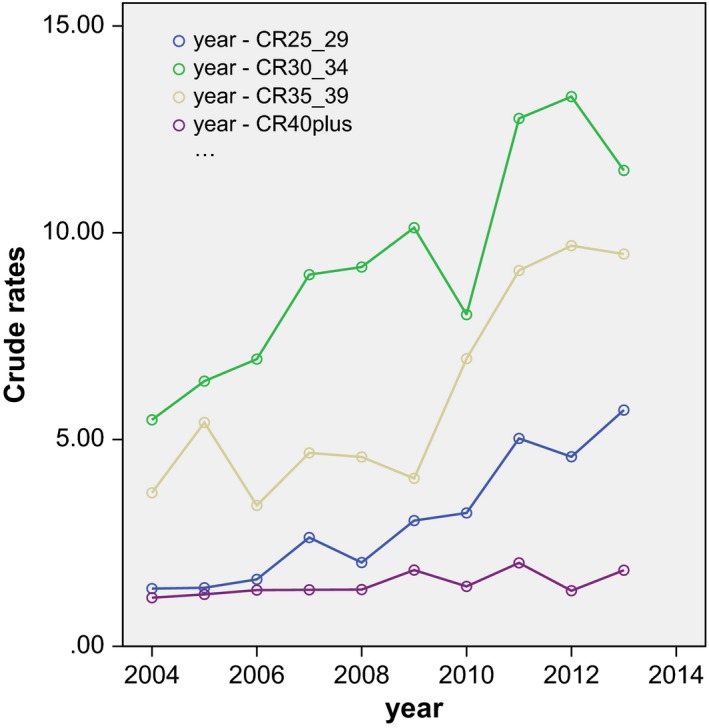
Crudes rates of adenocarcinoma in situ in the age categories 25–29, 30–34, 35–39, and over 40 in the years 2004–2013 with fit line.

After selection, a total of 1678 AIS patients were identified and included in this study. The mean incidence number of AIS over 10 years (2004–2013) was 167 cases per year and the mean ESR was 1.99 per 100,000 women years, compared to a mean ESR for adenocarcinoma of 1.59. The European standardized incidence rates of AIS showed a significant increase (*r* = 0.95; *P* = 0.00) in the years 2004–2013 with an EAPC of 9.20% (95% CI: 7.50, 10.92; *P* = 0.00) (Fig. 2 and Table 2). This increase is visible in the age groups from 25 up until 39 years (*P* = 0.00; Fig. 3). The crude and European standardized rates of adenocarcinoma in the years did not significantly increase in 2004 to 2013 (ESR: *r* = 0.33; *P* = 0.32) with an EAPC of 1.31 (95% CI: −0.66, 3.31; *P* = 0.24). In the 40+ age groups, the incidence of AIS showed only minor increment (*r* = 0.68; *P* = 0.03).

**Table 2:** EAPC 2004 to 2013, 95% confidence interval (95% CI) and ***P***‐value for ACA, AIS and AIS with (AIS+) and without (AIS‐) concomitant CIN

* *	EAPC (%)	95% CI (%)	*P‐*value
ACA	1.31	−0.66, 3.31	0.24
AIS	9.20	7.50, 10.92	0.00
AIS+	10.96	8.38, 13.60	0.00
AIS‐	6.93	5.06, 8.83	0.00

EAPC, estimated annual percentage change; CIN, cervical intraepithelial neoplasia; ACA, adenocarcinoma**;** AIS, adenocarcinoma in situ.

**Figure 4:**
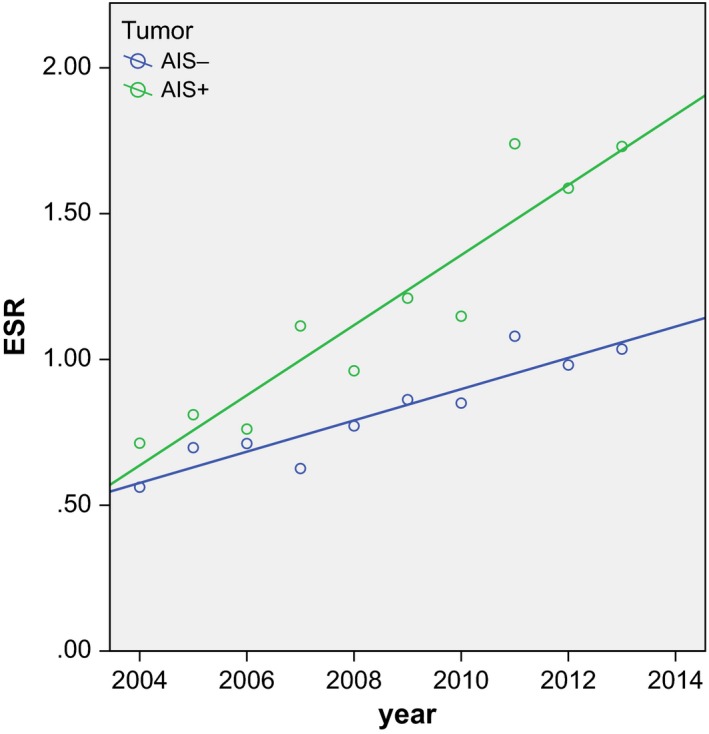
Scatterplot of the European Standardized Rates of adenocarcinoma in situ with (AIS+) and without (AIS‐) concomitant CIN in the years 2004–2013. AIS + *r* = 0.934 with *P* = 0.000 / AIS‐*r* = 0.928 with *P* = 0.000. CIN, cervical intraepithelial neoplasia.

There were 989 patients with AIS and concomitant CIN (58.9% of all AIS patients) and 689 patients with AIS but without concomitant CIN (41, 1%; *P* = 0.00). The mean age of AIS patients with concomitant CIN [36.0 years [SD: 7.8]) is significantly lower compared to AIS patients without CIN (40.2 years [SD: 9.6]; [*P* = 0.00]). AIS with concomitant CIN showed a slightly stronger increment in ESR (*r* = 0.93; *P* = 0.00) and EAPC (10.96%; 95% CI: 8.38, 13.60; *P* = 0.00) as compared to the group without concomitant CIN (*r* = 0.93; *P* = 0.00), which had an EAPC in between the rates for AIS and invasive adenocarcinoma (6.93%; 95% CI: 5.06, 8.83; *P* = 0.00) (Fig. 4 and Table 2). The EAPC of AIS with concomitant CIN was comparable to the rate of all AIS cases. When the correlation of the ESR of AIS with time was adjusted for the presence of CIN, the correlation was not significant (*r* = 0.57; *P* = 0.11).

## Discussion

The aim of this study was to explore the most recent incidence rates of cervical lesions in the Netherlands with emphasis on adenocarcinoma in situ. First we examined the European standardized incidence rates (ESR) and the estimated annual percentage change (EAPC) for invasive cancers and found that between 1989 and 2013 the incidence of cervical cancer, predominantly squamous cell carcinoma significantly decreased. The incidence of invasive adenocarcinoma remained relatively stable. However, when rates were broken down into two time periods, there was a difference. In 1989–2003, the incidence of all types of cervical cancer decreased, although nonsignificant for adenocarcinoma. During 2004–2013, the incidence of all cervical carcinomas remained stable. The trends in incidence of invasive cervical carcinomas in the Netherlands as found by Bulk et al. and de Kok et al. 8, 9 were confirmed in this study. The same trends are found worldwide 1, 4, 5, 6, 7. A possible explanation for this is the increased detection rate for CIN in the Netherlands, as found by Rozemeijer et al. 12 during 2000–2011. The authors contribute this increase to the implementation of liquid‐based cytology, but also an increased cervical cancer risk, which was confirmed by this study.

The main goal of this study was to investigate the incidence rates of adenocarcinoma in situ (AIS) of the uterine cervix in 2004–2013. Crude rates were calculated per 100,000 person years, using the absolute incidence of invasive carcinomas from the national cancer registry and data concerning AIS retrieved from PALGA, combined with population data from Statistics Netherlands. The crude rates for adenocarcinoma calculated by this method showed only minor variance from the numbers retrieved from IKNL. From these crude rates, the European standard rates (ESR) per 100,000 women years were determined.

AIS of the uterine cervix is considered as a precancerous lesion of invasive cervical adenocarcinoma. Convincing evidence includes (1) the fact that AIS is usually diagnosed in populations 10–20 years younger than those with invasive adenocarcinoma, (2) AIS frequently coexists with invasive adenocarcinoma on histological specimens, (3) records of cases of untreated AIS that precede invasive adenocarcinoma, and lastly (4) the discovery of similar high‐risk human papillomavirus (hrHPV) types in both AIS and adenocarcinoma 2. Therefore, one would expect that timely identification of AIS would decrease the incidence of invasive adenocarcinoma.

We found a total of 1678 cases of AIS between 2004 and 2013. In this period, the incidence of AIS significantly increased. Compared to this, invasive adenocarcinoma showed a slight but nonsignificant increase. The increase in AIS was predominant in women in the 25–39 age groups. This finding is not in accordance with the trends found by van de Nieuwenhof et al.^10^, who described a decrease in the adenocarcinoma in situ incidence with a stable adenocarcinoma incidence in the period of 1989–2003. The decrease in AIS incidence, as found by them was predominantly in the age groups 15–29, 45–49, and over 60. However, their method of data retrieval concerning AIS cases might have differed from the one used in this study. By using the Dutch national pathology database (PALGA) and scrutinizing all records with the specific terms mentioned as mentioned above, we are convinced that our dataset is more complete. This can partly explain the difference between this study and the study by van de Nieuwenhof 10.

In other countries worldwide, an increase in both the adenocarcinoma and adenocarcinoma in situ incidence was found 4, 7, 9. This is comparable to the trends found by us in this study, so it seems there are other factors at hand.

There are several possible explanations for the increase in AIS incidence found in this study. A more systematic screening for and treatment of CIN could have led to the incidental detection of AIS since glandular cervical lesions are often associated with high‐grade cervical intraepithelial neoplasia (CIN2‐3) 13, 14, 15, and these combined lesions have higher percentages of hrHPV compared to glandular atypia and adenocarcinoma in situ not associated with squamous lesions 13, 15. In our study, more than half of AIS patients had concomitant CIN and we confirmed that the increase in AIS rates was stronger in AIS cases with concomitant CIN.

The association of human papilloma virus (HPV) and cervical squamous cell carcinoma is well known and causal in nature. Squamous cell carcinomas are preceded by CIN 16 and over 90% of carcinomas harbor hrHPV, with HPV type 16 and HPV type 18 as the most common subtypes, with an overall predominance of HPV type 16 17. However, in endocervical columnar lesions, the role of hrHPV is less clear‐cut, although adenocarcinoma and squamous cell carcinoma share many of the same risk factors with the exception of smoking 18. HrHPV is detected in a majority of adenocarcinomas, with slightly lower frequencies, which vary between 70% and 98% 7, 17, 19, 20, 21, 22, 23, 24. In adenocarcinoma (in situ), there is a predominance of HPV types 16, 18 20, 25, 26, and 45 24. Furthermore, hrHPV incidence varies between adenocarcinoma subtypes, for instance, in minimal deviation adenocarcinoma, gastric type and clear cell adenocarcinomas, a significantly lower level of hrHPV infection was found 24, 25 or hrHPV was not detected at all 7, 20, 27, 28.

For AIS, the implementation of liquid‐based cytology and the cytobrush could also have contributed to the increased incidence we found in this study, comparable to the results for CIN as found by Rozemeijer et al. 12. Glandular lesions are often located deep in the endocervical canal and therefore more difficult to sample with the conventional Pap‐smear tools. Furthermore, in conventional Pap‐smears, the cytological features of AIS are subtle and often show overlap with the cytological features of CIN. In liquid‐based cytology, the sensitivity for glandular lesions is increased 29. This together with a better understanding of morphology and etiology and therefore a higher awareness by pathologists of glandular lesions, could lead to improved recognition of glandular lesions. This higher awareness could also have ensured better coding and reporting of AIS in the PALGA database. Furthermore, in 2007, hrHPV triaging was implemented in the Netherlands. This meant that the first repeat cytology after detection of a borderline lesion (ASC‐US of LSIL) is combined with hrHPV testing. This could have led to the detection of more precursor lesions, for instance concomitant squamous and glandular lesions.

As stated by Rozemeijer et al. 12, another explanation could be a change in risk factors. In the Netherlands, the HPV16 antibody seroprevalence has increased during adolescence and shifted to younger ages 30. This is probably due to a younger age at first intercourse. The same study also found a higher prevalence of HPV type 18, which is, as mentioned above, the biggest contributor in cervical adenocarcinoma. The increasing HPV prevalence in the population is also reflected in increasing incidence rates of other HPV‐related cancer types, such as anal and vulvar carcinoma 12. However, it is too early to see any effects of HPV vaccination, since this was introduced in the Dutch Nationwide Immunisation Programme in 2010 31, 32.

In the Netherlands, a nationwide screening program with the primary goal of lowering mortality and morbidity of cervical cancer by detecting and treating precancerous squamous cervical lesions is implemented. The Dutch nationwide screening program for cervical cancer invites women to participate at a 5 year interval which starts at age 30 and ends at age 60. In this study, most AIS lesions were detected around the ages when women were invited to take part in the screening program. The highest peak in incidence was around 19–30 years, which, when this group was further subdivided, could in majority be accounted for by 29‐year‐old women. Since women receive an invitation for screening in the year in which they turn 30, this peak can be explained by participation in the national screening program. Since this screening is more effective in detecting squamous intraepithelial neoplasia, it would stand to reason that most cases of AIS which were detected through the screening program would have a concomitant CIN. This was confirmed in this study, since the group of women with both AIS and CIN was significantly larger than the group of women with AIS lacking concomitant CIN.

This effect of screening could also be seen in the incidence levels of invasive cervical carcinomas. The incidence of all types of invasive cervical cancer showed two peaks (data not shown). The first peak was in the 35–39 year age group. This coincides with the second screening round in the Dutch nationwide screening program. The second peak was in the 70–74 year age group.

But despite the nationwide screening program in the Netherlands, there was an increase in AIS with stable rates of invasive adenocarcinoma. An increase in incidence of invasive adenocarcinomas as well as in situ adenocarcinomas has been shown in multiple countries worldwide, such as the Unites States, Korea, Sweden, and other European countries 1, 4, 7, 33. However, presumably with adequate screening and treatment of the precursor lesion of adenocarcinoma, one would expect a decrease in the incidence of invasive adenocarcinoma. Therefore, an increase in AIS rate (or an increase in AIS detection) could lead to a subsequent drop in adenocarcinoma incidence. This was not found in this study, and also other studies found the same phenomenon of cervical cancer screening seemingly failing to decrease adenocarcinoma incidence 7, 9, 31. Theoretically, it could be that this effect is not yet visible, since it takes approximately 10 years for AIS to progress into invasive adenocarcinoma 2. Or it is possible that part of the precursor lesions we are detecting would have shown regression on their own.

In conclusion, the trends in incidence rates of adenocarcinoma in situ and invasive carcinomas of the uterine cervix that are present worldwide are also found in the Netherlands. In the years 1989–2003, the incidence of all types of cervical cancer declined, while in 2004–2013, the incidence remained stable. This was accompanied by an increase in AIS incidence rates during 2004–2013, predominantly caused by cases of AIS with concomitant CIN.

## Conflict of Interest

None declared.
